# Reliability of an “At-Home” Method for Monitoring Resting and Reactive Autonomic Nervous System Activity in Children: A Pilot Study

**DOI:** 10.3390/children11070835

**Published:** 2024-07-09

**Authors:** Rachel Venn, Joseph M. Northey, Nenad Naumovski, Andrew McKune

**Affiliations:** 1 School of Rehabilitation and Exercise Sciences, Faculty of Health, University of Canberra, Canberra, ACT 2617, Australia; rachel.venn@canberra.edu.au (R.V.); joe.northey@canberra.edu.au (J.M.N.); nenad.naumovski@canberra.edu.au (N.N.); 2 Research Institute for Sport and Exercise, University of Canberra, Canberra, ACT 2617, Australia; 3 Functional Foods and Nutrition Research (FFNR) Laboratory, University of Canberra, Ngunnawal Land, Canberra, ACT 2617, Australia; 4 Department of Nutrition and Dietetics, Harokopio University, 17671 Kallithea, Attica, Greece; 5 School of Health Sciences, University of Kwazulu-Natal, Durban 3629, South Africa

**Keywords:** heart rate variability, autonomic nervous system, children

## Abstract

Background: Heart rate variability (HRV), an index of the functional status of the autonomic nervous system (ANS), provides an opportunity for early detection of ANS dysfunction. Lower resting, vagally related HRV parameters are associated with increased risk of physical and mental illness. External factors influencing the ANS, such as the testing environment, may impact the interpretation of HRV. This study’s main aim was to determine the reliability of HRV resting and reactivity tests performed at home with children aged 4–9 years. Methods: Fourteen healthy children (female *n* = 8) aged 6.8 ± 1.5 years participated. Two HRV tests were performed at home via online supervision 7 days apart using a Polar H10 heart rate monitor. The absolute and relative reliability of the pre-exercise resting (5 min) and sub-maximal exercise step test recovery (4 × 30 s segments) HRV time and frequency domains were calculated. Results: The Pearson correlation coefficients for day 1 versus day 7 for the vagal activity HRV domains (RMSSD log) at rest and in the first 30 s and 30–60 s of recovery indicated good-to-excellent relative reliability (r > 0.8, *p* < 0.01). Absolute reliability was moderate for the resting RMSSD log, with a coefficient of variation (CV) of 5.2% (90% CI: 3.9, 7.8%), high for the first 30 s of standing recovery, with a CV of 10.7% (90% CI: 8.2, 15.7%), and moderate for 30–60 s of recovery, with a CV of 8.7% (90% CI: 6.6, 12.9%). Conclusions: The findings of this pilot study indicate that the resting and exercise recovery HRV measures of vagal activity can be measured reliably at home in children. This represents a novel “at-home” protocol for monitoring ANS health and development in children.

## 1. Introduction

Early life and chronic stress in children can result in the development of social, emotional, cognitive, and behavioural challenges which are associated with prolonged activation and dysregulation of the physiological stress response [1]. Dysregulation of the physiological stress response may also determine the capacity to respond to future stressful conditions and negatively influence overall physical or psychological health [2,3,4].

The physiological response to stress is reflected by the interrelated activation of the hypothalamic–pituitary–adrenal axis (HPA axis) and the autonomic nervous system (ANS) [5]. Activation of the ANS in response to a stressor results in a rapid (in milliseconds) withdrawal of the parasympathetic nervous system (PNS) (mediated by decreased acetylcholine release) and a slower (in seconds) increase in sympathetic nervous system (SNS) activity (mediated by the release of epinephrine and norepinephrine) [6]. These changes can lead to an increase in the energy supply to respond to the demands of a challenging situation [7,8,9]. During “normal conditions”, when the stressor has dissipated, the ANS demonstrates flexibility and quickly transitions back to PNS dominance [10]. ANS dysfunction has been shown to occur prior to the observation of clinical diagnostic signs and symptoms. It is characterised by an imbalance between its two branches (the SNS and PNS), and represented by changes in heart rate variability (HRV) [11].

HRV is a measure of the variability in the duration (intervals) between consecutive heart beats [12,13] (R waves) over time. Variation occurs as a result of the SNS and PNS modulating the heart rate [14]. Children with poorer ANS flexibility (e.g., longer PNS withdrawal or recovery to the HRV baseline) are proposed to be at greater risk of mental illness (e.g., anxiety and depression) and cardiometabolic disease [15]. Several studies have proposed limited ANS responsivity in children being connected with different mental health conditions, such as anxiety [16]. With little evidence on assessing ANS activation alone in young children, this limits our understanding of the effects of psychosocial stress on physiological stress responses and their contribution to mental health risk conditions [17,18]. HRV provides an opportunity for early detection of ANS dysfunction in the identification of children at risk of developing physical and mental health issues [19]. HRV is widely used in studies on adult populations, but fewer studies have been conducted with children [20].

HRV is a noninvasive marker of autonomic cardiac regulation, with increased vagally mediated HRV at rest generally indicative of better health, aerobic fitness, self- regulatory capacity, and adaptability or resilience [21]. On the other hand, decreased resting vagally mediated HRV is indicative of poor health and worse outcomes among conditions such as cardiac arrhythmia, obesity, hypertension, type 1 and type 2 diabetes mellitus, and psychological disorders [22,23,24,25,26]. Higher levels of resting vagally mediated HRV are linked to the performance of executive functions, like attention and emotional processing, by the prefrontal cortex [27], but a lack of inhibition of frontocortical activity leads to undifferentiated threat responses to environmental challenges [19].

In addition to the resting measures of vagally mediated HRV, the magnitude of vagally mediated HRV reactivity or ANS flexibility in response to and recovery from a stressor (psychosocial or physical) has been identified as an independent and noninvasive marker of physical, behavioural, and emotional resilience [28]. Children with poorer ANS flexibility (i.e., longer PNS withdrawal or recovery to baseline HRV) are proposed to be at greater risk of developing mental health issues (anxiety and depression) [15,16] and cardiometabolic disease [15].

In addition, there are established links between cardiorespiratory fitness, mental illness, and cardiometabolic disease in both adults and children with lower cardiorespiratory fitness levels associated with reduced HRV reactivity, as well as influencing risk factors and short- and long-term health outcomes [29,30,31,32]. Therefore, monitoring resting HRV and HRV reactivity in young children is important, considering that rapid development of the ANS occurs early in life and is associated with health outcomes [33].

Heart rate variability is influenced by a wide range of variables, making it challenging to compare HRV findings across studies in children [34,35]. For instance, some participants may experience an increase in stress or anxiety when entering a clinic or laboratory environment. This will impact the physiological stress response [7] and may reduce the ability to obtain a valid resting HRV measure in children. Therefore, the methodology and study design are important to consider for valid and reliable HRV measures in children. There is limited research on the assessment of resting and reactivity HRVmeasures for understanding the effects of psychosocial stress on the physiological stress response in young children [17,18]. Therefore, to interpret the data accurately, this study included a healthy population. Whilst there are some guidelines which refer to obtaining reliable resting HRV measures in young children [20], there are currently no studies that have been conducted with participants in their home environments. Therefore, the aim of this study was to investigate the test-retest reliability of measuring HRV in healthy, young children in their home environments under resting conditions and in response to physical stress.

## 2. Materials and Methods

### 2.1. Participants

This study was conducted with 14 healthy children (male *n* = 6; female *n* = 8) between the ages of 4 and 9 years old (6.8 ± 1.5 yrs) (Table 1). The sex, date of birth, weight, body mass index (mass (kg)/height (m^2^)), and stature of each child were provided by the parents. The participants were classified as being of healthy weights based on their body mass indices (16.2 ± 2.3 kg/m^2^) (Table 1). Participation was voluntary, with written informed consent gained from the parents or guardians of each child. Ethical approval was granted by the University of Canberra Human Research Ethics Committee (UCHREC 1971).

Participants were excluded if they were taking medications (e.g., Adderall, Ritalin, cholinesterase inhibitors, or selective serotonin re-uptake inhibitors) that have been shown to impact the ANS [36,37,38]. Although there is limited research on these effects in children, these factors were excluded based on evidence in adults [39].

### 2.2. Study Design

The study used a test-retest reliability design to compare individual HRV results collected in the home environments before, during, and after a sub-maximal exercise test with children on two occasions 7 days apart. HRV data were collected using a Polar H10 heart rate sensor chest monitor (Polar Electro Oy, Kempele, Finland) and uploaded onto the EliteHRV© app version 5.5.8. The lead researcher observed and instructed testing through the Coviu© Global Pty Ltd. (Sydney, Australia) telehealth platform. Remote supervision by the investigator allowed for a detailed record of observations of the child, for example, being unable to stay physically still during the resting and recovery phases, being unable to maintain rhythm or keep to the beat of the metronome during the test, or needing to hold a parent’s hand during testing. This ensured that any deviation from the protocol was documented for inclusion in the discussion.

### 2.3. Study Procedures

#### 2.3.1. Familiarisation Session

One familiarisation session was conducted with the aim to reduce stress-related changes in physiological activity prior to data collection, which may be especially true among infants and young children, and to allow participants to acclimate to their surroundings and the physiological sensors prior to HRV recording [40,41]. Parents were informed of the step height specific to their child, which accounted for the individual height (stature) and a hip angle of 73°, thus standardising the work load [42] (Figure S1, Table S1).

Parents were guided with instructions for fitting the heart rate monitor and taking a test reading to obtain the R-R interval data for HRV analysis. Any troubleshooting, such as tightening the strap to reduce movement, was important to ensure a quality reading was received. Each child completed a practice step test and HRV reading, with the lead investigator observing and providing encouragement and guidance to the child.

#### 2.3.2. Resting HRV Measurement

The parents were instructed to wet the electrodes on the reverse side of the Polar H10 chest strap and then place them around the participant’s chest, with the HR monitor placed on the xiphoid process of the sternum. The parent was instructed to ensure that the chest strap’s fitting was firm (not slipping). In line with the protocol established by Gerardo et al., and with methodological considerations applied based on the Task Force Guidelines [40] and McGrath and Weiner’s recommendations [43], a short-term recording of 5 min was performed under physiologically stable conditions (resting) and processed with the time and frequency domain methods. Participants were instructed to sit still and quietly during the resting recording, but they were allowed to read or be read to or watch a non-stimulating show of their choice to assist with ensuring they were physically still and quiet during the recording. Behaviours such as humming or talking, changing breathing patterns, and movement may influence HRV readings [43].

#### 2.3.3. Sub-Maximal Exercise Test HRV Measurement

The sub-maximal exercise test was divided into three phases.

The initial 30 s stabilisation period allowed for an effect on sympathetic activation, which was influenced by gravity and body mass, to be the same as that during the step test. The stabilisation period was followed immediately by 3 min of stepping at 88 clicks/min.

The 3 min step test protocol we selected was validated in subjects between 6 and 47 years of age, and it is easy to perform and tolerated well by school-aged children [44]. The test allows for estimation of the cardiorespiratory fitness of children and monitoring changes in the cardiovascular system in response to sub-maximal exercise [45]. The protocol was designed to ensure equal work efficiency and limit early muscle fatigue and exhaustion [44,46]. The 3 min duration was selected based on Task Force Guidelines, which suggest a minimum of 2 min when assessing either high-frequency or low-frequency HRV parameters [40].

At the end of 3 min of stepping, the participant was instructed to stand extremely still and be quiet for a further 2 min. HRV recovery in the phase immediately after the 3 min step test provided an indication of the speed of vagal recovery, and this is paramount to physical and psychiatric health outcomes [12].

The age-predicated maximal heart rate (MHR) percentage was used to indicate how “stressful or intense” the step test was, with the Tanaka equation (MHR = 208 − 0.7 × age) shown to be the most accurate method when determining age-predicted MHR in children [47].

The termination criteria of the step test were based on the recommendations of the ACSM Guidelines for Exercise Testing and Prescription, 8th edition [48]. These include the participant’s desire to stop, fatigue, shortness of breath, leg cramps, wheezing, dizziness, nausea, abnormal HR (too fast or too slow), and if the stepping rhythm was three consecutive beats slower than the metronome.

### 2.4. Heart Rate Variability Assessment Methods

The Polar H10 heart rate sensor chest monitor has proven validity and reliability in assessing R-R intervals during rest and physical exercise [39,49,50,51,52,53,54]. The heart rate variability standards of measurement paper by the Task Force of the European Society of Cardiology and the North American Society of Pacing and Electrophysiology [55] recommends the use of 250–500 Hz or higher for the sampling frequency for HRV measurements, although this depends on whether the time or frequency domain parameters are used. The Polar H10 samples the raw ECG signal at 1000 Hz, but after postprocessing, this signal is downsampled to 130 Hz. Studies have shown that a lower sampling frequency is still acceptable for time domain analysis (e.g., RMSSD), which we used in the current study. Specifically, Kwon et al. (2018) reported that a 250 Hz sampling frequency would be acceptable for HRV analysis for both frequency and time domain analysis. However, when frequency domain analysis is not required, a 100 Hz sampling frequency would also be acceptable for time domain analysis (e.g., RMSSD) [56]. Another paper by Lee et al. (2022) directly compared ECGs downsampled from 2000 Hz to 125 Hz and suggested that the 125 Hz ECG was accurate even for frequency domain measures [57].

Once downloaded, the raw R-R interval data were exported from the EliteHRV© app as a text file to Kubios HRV software (version 3.5.0, Biosignal Analysis and Medical Imaging Group, Department of Physics, University of Kuopio, Kuopio, Finland). The Kubios program complies with guidelines recommended by the Taskforce of the European Society of Cardiology and the North American Society of Pacing and Electrophysiology standards for the measurement of HRV [40]. Once the raw R-R intervals were imported into the Kubios program, the software automatically analysed the following HRV time and frequency parameters: the R-R intervals (interbeat intervals), mean heart rate (HR), standard deviation of normal-to-normal intervals (SDNN), mean square root differences of the standard deviation (RMSSD), number of pairs of successive R-R intervals that differed by more than 50 ms (NN50), and percentage of beats that changed more than 50 ms from the previous beat (pNN50) in the time domain and low frequency (LF (ms^2^, log, %, nu)), high frequency (HF (ms^2^, log, %, nu)), total power (ms^2^, log), and low-frequency-to-high-frequency ratio (LF:HF ratio (nu)) in the frequency domain. Power spectral analysis was conducted using the AR algorithm as it generates a better resolution, especially for short-term HRV measurements [58,59], and it was recommended by the taskforce [40].

The SDNN has been shown to reflect the parasympathetic, sympathetic, and circadian influences of cardiac activity, while the RMSSD and pNN50 reflects the parasympathetic influence [43]. Several studies have demonstrated that the RMSSD is the most consistent HRV domain for reflecting ANS activity at rest and reactivity, especially in field-based studies [13,17]. Heart rate variability measures are often observed to be non-normally distributed, and therefore data transformation (Ln) was applied to the absolute RMSSD in the present study [60]. As mentioned previously, the resting state HF HRV is a direct indicator of cardiac vagal or parasympathetic activity [61,62] and is also associated with respiratory influences [63,64,65,66] while LF HRV reflects an interaction of sympathetic and parasympathetic activity [67], and the LF:HF ratio is proposed to describe overall autonomic modulation [43]. The time domain variables, particularly RMSSD, are the least impacted by breathing frequency of the participants compared with the frequency domain variables [68].

The time and frequency domain HRV measures and data inclusion and exclusion criteria were guided by previous research [43]. Prior to analysis, R-R interval data were manually corrected using the following guidelines. If a significantly higher R-R interval (representing an ectopic beat) was identified, then that beat was deleted and replaced with the average of the two adjacent R-R intervals [40,69], and if a significantly lower value (representing a missed beat) was noted, then that R-R interval was deleted and replaced with the previous R-R interval [35]. To ensure the integrity, quality, and interpretability of the HRV data, all individual participant data included for the final analysis did not exceed 20% of the participants’ recordings [69].

### 2.5. Statistical Analysis

Analyses were conducted using IBM SPSS Statistics version 28.0 (IBM Corp., Armonk, NY, USA) and a custom MS Excel version 16 (Microsoft Corp., Redmond, WA, USA) spreadsheet [70]. A minimum sample size of *n* = 13 was required to achieve statistical significance, based on a Pearson correlation coefficient (r) of 0.70, for an alpha set at 0.05 and a minimum power of 90% [71]. The data were summarised using descriptive statistics (mean ± SD). Using the predicted $\dot{V}$O_2_max for each participant, a comparison of the intensity of the two step tests (day 1 versus day 7) was performed using a paired *t*-test. Significance was set at *p* < 0.05. To determine the test-retest reliability of the resting (5 min) and recovery (2 min) phase divided into 4 30 s intervals (0–30, 30–60, 60–90, and 90–120), the HRV readings for day 1 versus day 7 and relative (Pearson correlation coefficient) [72] and absolute reliability (typical error of measurement (TEM) and coefficient of variation (CV)) were calculated using the procedures described by Hopkins, who deemed these variables sufficient to characterise the reliability of a measure [73]. The reliability of the HRV time and frequency domain parameters were calculated for the 5 min resting phase, whilst the reliability of only the time domain HRV parameters was calculated for the recovery phase, as the duration was less than 5 min. The calculations were able to identify within-subject variation, indicating the extent to which repeated resting and recovery HRV measures varied for the participants when compared 7 days apart. The CV was interpreted as low, moderate, or high if it was <5%, 5–10%, or >10%, respectively [74]. The Pearson correlation coefficient (r) value’s interpretation followed the guidelines of <0.5 being poor, 0.5–0.75 being moderate, 0.75–0.9 being good, and >0.9 being excellent [74].

## 3. Results

A total of 14 participants with complete data sets were included in the analysis. The means and standard deviations for age, height, weight, BMI, and age-predicted MHR percentage achieved for both step tests and the predicted $\dot{V}$O_2_max are displayed in Table 1. An exercise intensity for the 3 min step test at a cadence of 88 clicks/min, or moderate intensity exercise, resulted in greater than 60% age-predicted MHR [47] (Table 1). The predicted $\dot{V}$O_2_max (Table 1) indicates that the participants’ cardiorespiratory health was average for their ages [75]. Based on the paired *t*-test results, there was no significant difference between the predicted $\dot{V}$O_2_max values for day 1 versus day 7, indicating the same physiological performance for both days.

The absolute reliability, or the reliability of reproducing the measure, for the resting state was low to moderate for the R-R intervals (CV 0.2%; 90% CI: 0.1, 0.3%); RMSSD absolute (CV 3.1%; 90% CI: 2.4, 4.7%), and RMSSD log (CV 5.2%; 90% CI: 3.9, 7.8%) (Table 2). Resting for day 1 versus day 7 indicated good-to-excellent relative reliability (r) for the R-R intervals, RMSSD absolute, and RMSSD log (Table 2).

In the first 30 s post exercise recovery, there was moderate absolute reliability for the R-R intervals (CV 4.4%; 90% CI: 3.3, 6.5%) and high absolute reliability for the RMSSD log, with a CV of 10.7% (90% CI: 8.2, 15.7%) (Table 3). The first 30 s of recovery post step test on day 1 versus day 7 indicated good-to-excellent relative reliability for the R-R intervals and RMSSD log (r > 0.8, *p* < 0.01) (Table 3).

From 30 to 60 s post exercise recovery, the absolute reliability was moderate for the R-R interval (CV 5.9%; 90% CI: 4.5, 8.8%) and RMSSD log (CV 8.7%; 90% CI: 6.6, 12.9%) (Table 3). From 30 to 60 s of recovery for day 1 versus day 7, the R-R interval and RMSSD log relative reliability continued to be good to excellent (r > 0.8, *p* < 0.01) (Table 3).

From 90 to 120 s post exercise recovery, the absolute reliability for both the R-R interval and RMSSD log decreased (Table 3). From 90 to 120 s, the relative reliability for the R-R intervals was still moderate to good, although it decreased for the RMSSD log (Table 3).

## 4. Discussion

Heart rate variability is a validated tool for monitoring cardiac vagal activity [14,15], and it has been proposed to be used for early detection of disrupted ANS function, which is indicative of a risk of developing physical and mental illness. However, the incorporation of HRV monitoring to detect ANS disruptions in children is not a standard screening practice within the community, despite the method being noninvasive, inexpensive, and accessible. This study aimed to investigate the reliability of measuring HRV in children in their home environments, under resting conditions, and in response to physical stress. The main findings were that the vagally related HRV domains (RMSSD log) at rest and in the first 30 s (as well as 30–60 s) of recovery demonstrated good-to-excellent relative reliability (r > 0.8, *p* < 0.01). Absolute reliability was moderate for the resting RMSSD log, with a CV of 5.2% (90% CI: 3.9, 7.8%), high for the first 30 s of standing recovery, with a CV of 10.7% (90% CI: 8.2, 15.7%), and moderate for 30–60 s of recovery, with a CV of 8.7% (90% CI: 6.6, 12.9%). The results suggest that the resting and sub-maximal exercise recovery HRV time domain measures of vagal activity can be measured reliably at home in children aged 4–9 years. This study provides a novel protocol for monitoring ANS health and development in children in communities.

Some of the confounding factors that may impact the reliability of HRV measures include the participant’s sex, age, weight, and height [43,76]. Males are likely to have a greater sympathetic dominance relative to females during childhood development (related to changes in sex hormone concentrations), which may have an effect on their blood pressure and heart rate [34,35]. Sex differences in HRV reliability may exist among both adults and children. However, studies on children examining biological sex differences in HRV reliability are rare. Changes in HRV across developmental periods may influence the reliability of data collected from the age demographic of the study participants. Similar to the poor reliability found in the present study relating to the HRV frequency domain parameters, previous studies have observed lower test-retest HRV HF reliability for samples spanning from 2 months to 5 years of age [77], from 5 to 14 years of age [78], and among toddlers compared with children [79]. Based on the current results and previous research, it seems that the time domain HRV measures representing vagal activity are more reliable when testing children in their home environments.

The limitations of HRV analysis include a lack of standardisation of protocols and data measurement durations [43,80] and poor knowledge of the interpretation of the results found across various frequency bands [81]. The HRV measurement standards [40,41] indicate that HRV reliability measures are sensitive to specific methodological decisions, including the study protocol, sample characteristics, ECG signal acquisition, and preliminary processing and HRV analysis. However, there remains wide variability in study methodologies and a lack of accountability for differences in paediatric HRV measurements [82]. The present study attempted to provide a novel “at-home” HRV resting and reactivity protocol that could be built upon in future studies investigating the ANS health of children without the need for them to visit laboratory or clinic settings. The home environment provides a unique location to minimise variables that may influence a child’s HRV measurements, including circadian rhythms and stress. Circadian variations in HRV have been observed in children due to SNS dominance peaking just after awakening and withdrawing during the day, while PNS dominance becomes augmented throughout the night, reaching its peak before awakening [83,84,85]. Measuring HRV in the home environment has the benefit of reducing stress that may be felt upon arrival at a laboratory or clinic [7] and also allows for greater recruitment potential and increased access to those who live long distances from testing facilities [86].

To optimise test-retest reliability, consideration was given to behaviour that affects ANS physiology, such as exercise, circadian effects, and postural changes [87,88]. Due to the age range of the participants in this study, it was not feasible to eliminate physical activity participation, and therefore the time of day and postural changes were factored in as variables to account for when recording HRV measures. Resting baseline HRV measures were conducted with the participants in a seated position to allow for PNS dominance, with standing positions observing SNS dominance [65,89]. It is important to note that changes in sympathovagal balance across childhood development [34] will influence HRV, therefore becoming a further factor to consider when investigating HRV reliability. Resting HRV measurement was performed using a short-term (5 min) daytime measure of the time and frequency domain HRV protocol, which has shown moderate reliability in child and adolescent studies (Z = 0.62, r = 0.55) [43]. A 5 min baseline or resting recording is generally accepted as a standard in short-term studies [40,41], and the comparison of identical-length recording durations with a test-retest period of 7 days selected as a shorter interval length (less than two weeks) is more reliable than one that lasts months or years, especially with age-related changes in autonomic development increasing HRV [90]. The participants were asked to complete testing before 11:00 a.m. to account for potential circadian effects and to minimise exposure to daily stress [91].

The measurement of maximal oxygen uptake ($\dot{V}$O_2_max) during maximal exercise is the most accurate way to measure cardiovascular fitness, but limitations exist in attempting this with young children in a home environment [92]. These include requiring assistance during testing to ensure safety, cooperation, and motivation from the individual as well as the individual not being accustomed to the testing protocol and being unfamiliar with perceiving and coping with fatigue [93]. An alternative test of cardiovascular fitness is the sub-maximal exercise test [92]. Age- and gender-specific reference ranges have been published for the sub-maximal exercise heart rate, thus enabling the assessment and monitoring of sub-maximal exercise-induced changes in the cardiovascular system and, consequently, prediction of the $\dot{V}$O_2_max [45]. From the present results, it can be seen that sub-maximal exercise testing is a practical physical stress test for measuring ANS flexibility in the home environment [94], with its advantages including no elaborate, expensive testing equipment and being easy to administer. Potential limitations include an inconvenient protocol for testing highly fit individuals and the duration of the test resulting in younger subjects being less adherent to the prescribed intensity [45]. Research investigating the associations between health, fitness, and ANS flexibility in children is still relatively limited [95], but it would provide invaluable risk identification data for health outcomes related to physical and mental illness [45,96].

A limitation of this study includes its small sample size (*n* = 14). However, while more participants would have been beneficial, this number is still acceptable as a minimum sample size of *n* = 13 was required to achieve statistical significance, based on correlations of 0.70 for an alpha value set at 0.05 and a minimum power of 90% [71]. Additional limitations include previous activity levels, sleep, mood and behaviour, and breathing rate variations, even with the instruction to “breathe normally”. During the resting phase of the testing, some children fidgeted, wiggled, coughed, and talked when instructions to limit movement and talking were provided. However, the impact of these on the HRV measurements were investigated through inspection of the raw interbeat interval data. Any artefacts were manually corrected based on standardised protocols [35,40,62]. Distractions in the home environment such as siblings and pets were accounted for by ensuring they were kept out of the testing space or had a parent or carer minding them. Although we did collect age and gender data, we did not confirm the prepubertal status to account for the impact and effect of child development relating to sex hormone concentrations on HRV, therefore presenting a limitation. Future studies may benefit from using systems such as the Tanner staging score [97] to identify sexual maturity ratings and address this limitation. The parent or carer was asked to fit the HR monitor as guided by instruction from the researcher through a video connection, but children with small torsos made correct fitting of the heart rate strap challenging, affecting the data collection. This was accounted for through manual correction of the interbeat interval data [35,40,62]. Another limitation is the lack of analysis of the acceleration capacity, which is a measure of HRV and an indicator of ANS function. This parameter will be analysed in a future study to determine the “vagal threshold” [98], which will be used to investigate its value for exercise prescription for children aged 4–9 years. Whilst the Polar H10 uses a sampling frequency that is potentially acceptable for HRV time domain analysis, it is not a licensed medical device, and decisions made regarding the autonomic nervous system and child health would require further in-depth exploration at a clinic or laboratory.

## 5. Conclusions

The present study developed and investigated the reliability of a protocol to measure resting and exercise reactivity HRV in young children and monitor and track their health without the requirement to attend in-person clinic or laboratory appointments. The use of commercially available HRV measurement systems to monitor health and performance is growing in communities. However, there remains a lack of understanding of the confounding variables and appropriate testing protocols, as well as the interpretation of HRV parameters. The findings from this study provide guidance on how to obtain reliable resting and reactivity measures in children 4–9 years of age using the commercially available Polar H10 heart rate monitor and EliteHRV© HRV analysis application. At-home protocols for resting and reactivity HRV can be used as screening tools for identifying altered ANS functioning, which is indicative of a risk for developing physical and mental illness in young children. Future studies should determine the signs and symptoms of physical and mental illness which HRV is related to in children and the effects of therapeutic interventions.

## Figures and Tables

**Table 1:** Descriptive data of male and female healthy participants (mean ± SD).

		*n* = 14	Males (*n* = 6)	Females (*n* = 8)
Age (years)		6.9 ± 1.6	7.0 ± 1.9	6.8 ± 1.4
Height (m)		1.3 ± 0.1	1.3 ± 0.1	1.3 ± 0.1
Mass (kg)		27.7 ± 5.8	28.6 ± 5.5	27.0 ± 6.2
BMI (kg/m^2^)		16.2 ± 2.3	16.3 ± 1.4	16.1 ± 2.9
Percentage of age-predicted MHR during step test	Day 1	61.0 ± 3.7	58.3 ± 3.8	63.2 ± 1.6
	Day 7	62.9 ± 4.6	61.3 ± 4.9	64.1 ± 4.3
Predicted $\dot{V}$O_2_max (mL/kg/min) from 3 min step test	Day 1	38.1 ± 5.6	37.4 ± 6.3	38.7 ± 5.3
	Day 7	38.1 ± 5.6	37.4 ± 6.3	38.7 ± 5.3

**Table 2:** Reliability indices for frequency and time domains in resting HRV parameters.

	Day 1 vs. Day 7 Resting HRV		
	Absolute Reliability		Relative Reliability
HRV Parameter	TEM	TEM (%)	r
LF/HFnu	0.38 (0.29–0.56)	63.3 (48.3–93.3)	0.48 (0.03–0.77)
HFnu	13.71 (10.45–20.36)	20.6 (15.7–30.6)	0.20 (−0.28–0.60)
LFnu	11.02 (8.40–16.37)	34.9 (26.7–51.9)	0.39 (−0.08–0.72)
R-R intervals (ms)	1.41 (1.07–2.08)	0.2 (0.1–0.3)	0.86 (0.66–0.95)
SDNN (ms)	13.95 (10.63–20.72)	15.4 (11.8–22.9)	0.78 (0.50–0.91)
RMSSD (ms)	1.41 (1.07–2.09)	3.1 (2.4–4.7)	0.86 (0.66–0.95)
RMSSD log (ms)	0.24 (0.18–0.36)	5.2 (3.9–7.8)	0.99 (0.99–0.99)

HRV = heart rate variability. Frequency domain: LFnu = low-frequency normalised units, LF/HFnu = low-frequency-to-high-frequency ratio in normalised units. Time domain: R-R intervals = interbeat intervals, SDNN = standard deviation of normal-to-normal intervals, RMSSD = root mean squared differences of the standard deviation. Reliability: r = Pearson’s correlation coefficient expressed as a mean (90% CI), TEM = typical error of measurement, TEM% = typical error of measurement as a percentage, both expressed as means (90% CI).

**Table 3:** Reliability indices for HRV time domain parameters at 0–30, 30–60, 60–90, and 90–120 s standing recovery following 3 min of sub-maximal exercise test.

	Day 1 vs. Day 7		
	0–30 s Recovery		
	Absolute Reliability		Relative Reliability
**HRV parameter**	**TEM**	**TEM (%)**	**r**
R-R intervals (ms)	22.82 (17.40–33.90)	4.4 (3.3–6.5)	0.87 (0.69–0.95)
SDNN (ms)	8.99 (6.86–13.36)	27.5 (20.9–40.8)	0.74 (0.42–0.89)
RMSSD (ms)	10.49 (8.00–15.58)	34.8 (26.5–51.6)	0.72 (0.38–0.08)
RMSSD log (ms)	0.33 (0.25–0.50)	10.7 (8.2–15.7)	0.78 (0.51–0.91)
pNN50 (%)	5.71 (4.35–8.48)	69.6 (53.0–103.4)	0.68 (0.32–0.87)
	**30–60 s Recovery**		
**HRV parameter**	**TEM**	**TEM (%)**	**r**
R-R intervals (ms)	37.50 (28.59–55.70)	5.9 (4.5–8.8)	0.86 (0.65–0.94)
SDNN (ms)	18.46 (14.08–27.43)	35.1 (26.7–52.1)	0.46 (−0.00–0.760)
RMSSD (ms)	25.37 (19.34–37.68)	46.8 (35.7–69.6)	0.56 (0.14–0.81)
RMSSD log (ms)	0.33 (0.25–0.49)	8.7 (6.6–12.9)	0.75 (0.44–0.89)
pNN50 (%)	11.13 (8.48–16.53)	52.1 (39.7–77.4)	0.71 (0.38–0.88)
	**60–90 s Recovery**		
**HRV parameter**	**TEM**	**TEM (%)**	**r**
R-R intervals (ms)	45.11 (34.39–67.01)	6.6 (5.1–9.8)	0.69 (0.34–0.87)
SDNN (ms)	24.06 (16.05–31.28)	36.5 (24.4–47.5)	0.44 (−0.01–0.75)
RMSSD (ms)	24.28 (18.51–36.07)	37.2 (28.4–55.3)	0.69 (0.34–0.87)
RMSSD log (ms)	0.38 (0.29–0.56)	9.5 (7.2–13.9)	0.63 (0.23–0.84)
pNN50 (%)	11.43 (8.72–16.98)	39.9 (30.4–59.2)	0.62 (0.22–0.84)
	**90–120 s Recovery**		
**HRV parameter**	**TEM**	**TEM (%)**	**r**
R-R intervals (ms)	56.87 (42.52–88.19)	8.6 (6.4–13.3)	0.78 (0.47–0.92)
SDNN (ms)	18.54 (14.00–28.09)	35.7 (26.9–54.1)	0.28 (−0.22–0.67)
RMSSD (ms)	23.69 (17.89–35.90)	46.3 (35.0–70.2)	0.47 (−0.01–0.77)
RMSSD log (ms)	0.44 (0.33–0.67)	11.8 (8.9–18.0)	0.54 (0.08–0.77)
pNN50 (%)	14.46 (10.93–21.91)	61.6 (46.6–93.3)	0.56 (0.11–0.82)

HRV = heart rate variability. Time domain: R-R intervals = interbeat intervals, SDNN = standard deviation of normal-to-normal intervals, RMSSD = root mean squared differences of the standard deviation, pNN50 = percentage of beats that changed more than 50 ms from the previous beat. Reliability: r = Pearson’s correlation coefficient, expressed as a mean (90% CI), TEM = typical error of measurement, TEM% = typical error of measurement as a percentage, both expressed as means (90% CI).

## Data Availability

The data presented in this study are available on request from the corresponding author. The data are not publicly available due to current ethics approval.

## Supplementary Materials

The following supporting information can be downloaded at https://www.mdpi.com/article/10.3390/children11070835/s1. Figure S1: The height that the foot will rise (*H_f_*) when the hip is flexed at angle *θ* can be determined using the relationship *H_f_* = (*h*) (1 − cos*θ*), where *h* is the length of the femur. Table S1: Ratio of femur length to stature in children. The ratio is designated as Lf and is used in the step height equation to determine the platform height. Refs. [99,100,101,102] are cited in Supplementary Materials
