# Supplementary material for: Reliability of an “At-Home” Method for Monitoring Resting and Reactive Autonomic Nervous System Activity in Children: A Pilot Study
Source: Children (Basel). 2024 Jul 9;11(7):835. doi: 10.3390/children11070835 (PMC11275235; doi:10.3390/children11070835)
Supplement: Supplementary file 1 [file children-11-00835-s001.zip › children-3045761-supplementary.pdf]

Figure S1. The height that the foot will rise *(H_f)* when hip is flexed at angle Θ can be determined using the relationship $H_f=(h)(1-\cos\Theta)$ where *h* is length of femur [1].

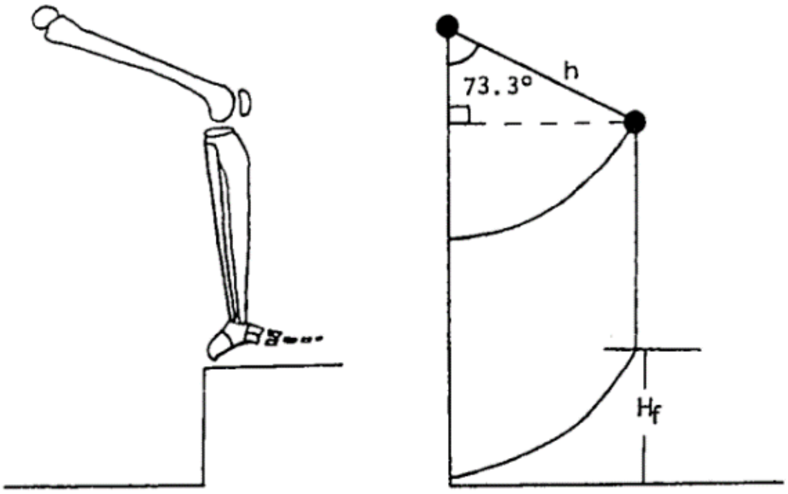

[1] Francis, K.; Feinstein, R. A simple height-specific and rate-specific step test for children. *South. Med. J.* **1991**, *84*(2), 169-74; https://doi.org/10.1097/00007611-199102000-00005

Table S1. Ratio of femur length to stature in children. The ratio is designated Lf and is used in the step height equation to determine the platform height.

Step height (cm) = (Lf x Ih) (1 - cos 73 degrees)
where Lf = Ratio of femur length to height and Ih = Subjects height (cm)

Age (years)	Gender	Lf = estimates based on growth rates during 4-6 year [1]
4 - 5	M	*0.255 note: this figure is estimated based on the male 6-9 ratios*
	F	*0.256 note: this figure is an estimate based on the female 6-9 ratios*
		**Lf = Ratio of femur length to height [2,3]**
6 – 8	M	0.257
	F	0.258
9	M	0.259
	F	0.262
10	M	0.263
	F	0.264

[1] Baumgartner, R.N.; Roche, A.F.; Himes, J.H. Incremental growth tables: supplementary to previously published charts. *Am. J. Clin. Nutr.* **1986**, *43*(5), 711-22; https://doi.org/10.1093.ajcn/43.5.711
[2] Anderson, M.; Green, W.T. Lengths of the femur and tibia: Norms derived from orthoroentgenograms of children from five years of age until epiphysial closure. *Am. J. Dis. Child.* **1948**, *75*(3), 279-90; https://doi.org/10.1001/archpedi.1948.02030020291002
[3] Green, W.T.; Wyatt, G.M.; Anderson, M. Orthoroentgenography as a method of measuring the bones of the lower extremities. *J. Bone Joint Surg. Am.* **1946**, *28*, 60-5.
